# Potential Role of the Moderna COVID-19 Vaccine in Enoxaparin’s Effects on Liver Functions

**DOI:** 10.7759/cureus.24018

**Published:** 2022-04-10

**Authors:** Omar Rafa, Iger Ostreni, Eric J Basile, Avneet Singh

**Affiliations:** 1 Internal Medicine, University at Buffalo, Buffalo, USA; 2 Medicine, Touro College of Osteopathic Medicine, New York City, USA; 3 Medicine, Touro College of Osteopathic Medicine, Staten Island, USA; 4 Internal Medicine, University of Florida College of Medicine, Gainesville, USA; 5 Internal Medicine, Cooper University Hospital, Camden, USA

**Keywords:** covid19 enoxaparin, covid-19 and liver, abnormal liver function test, liver function, enoxaparin, covid-19 moderna vaccine

## Abstract

Enoxaparin is commonly used for prophylaxis as an anticoagulant in hospital settings. Although enoxaparin has been known to cause many minor adverse reactions, hepatocellular injury is one of the rare side effects which can impact clinical course, marked by an asymptomatic rise in liver function panel tests. In this paper, we not only delineate the relationship between enoxaparin-induced hepatocellular injury but also associate it with fevers that have not been previously documented. Furthermore, we posit the Moderna COVID19 vaccine as a potential contributor to this outcome. We hypothesize that the link between enoxaparin and hepatic injury is possibly due to the inflammatory state, which may be augmented by the vaccine.

## Introduction

Since the 1970s, low-molecular-weight heparins (LMWH), such as enoxaparin, have proved their benefit over unfractionated heparins (UH) in the use of anticoagulation [1]. Their preferred use comes from the fact that they inhibit the clotting Factor X while not having as much of an extensive bleeding profile as UH does [1]. In fact, one study from the American Society of Hematology found that, when used for prophylactic reasons, enoxaparin specifically has around 1.7% incidence in causing major bleeds; whereas UH had an incidence of 3.5% [2]. However, there are definitely adverse effects of enoxaparin which include but are not limited to: injection site swelling, irritation, pain, bruising, and redness as well as fatigue, fever, and hepatocellular injury [1]. For the purpose of our case, we will limit the discussion to the last-mentioned effect.

The Food and Drug Administration’s (FDA) Adverse Event Reporting System states that hepatic injury secondary to enoxaparin accounts for 4% of all adverse events of this medication [3]. Enoxaparin’s role in causing hepatocellular injury is well documented in the past. Such cases often report the injury occurring within a week of starting therapy. The liver transaminases can even rise up to five times the normal upper limit. The prognosis is great following discontinuation of the enoxaparin, with transaminase levels normalizing promptly [4]. Symptoms like fever, fatigue, and pain are uncommon in most cases.

Other factors that predispose the liver to injury by enoxaparin should be evaluated. One such predisposition that may potentially exist is the Moderna COVID-19 vaccine. Regarding the Moderna (mRNA-1273) vaccine, reactions reported in clinical trials include pain/swelling/erythema at the injection site, fatigue, headaches, myalgia, arthralgia, chills, nausea/vomiting, axillary swelling/tenderness, fever. This vaccine creates a state of inflammation in the body, which may potentially play a role in deeming patients on enoxaparin more prone to hepatocellular injury from this specific anticoagulant [5]. There are patients who have been reported to have developed inflammatory conditions such as myocarditis following the same vaccine administration [6]. We present a case, in which, a 77-year-old male who had undergone a second dose of the vaccine developed hepatocellular injury following administration of enoxaparin.

## Case presentation

Our case is a 77-year-old male with a past medical history of dementia, peripheral neuropathy, bilateral knee osteoarthritis, and recurrent history of mechanical falls who presented to the emergency department (ED) with a complaint of mechanical fall four days prior and a low-grade fever following the second dose of the Moderna COVID-19 Vaccine (mRNA-1273), which he received five days previously. In the ED, he was found to be febrile at 100.7˚F with worsening weakness. All of his other vitals were within normal limits with a blood pressure of 118/78 mm Hg, a heart rate of 85 beats per minute, and a respiratory rate of 16 breaths per minute. He was also noted to have supra-pubic tenderness on physical examination. He complained of intermittent dysuria of one week's duration, for which he had an abdominal CT scan was done that showed no evidence of acute abnormalities. Cardiac workups including chest x-ray and EKG were both negative. Sepsis workup and viral screen were negative (including COVID-19 and Influenza). He was given 975 mg of acetaminophen, 1 liter of normal saline bolus, and admitted for fever workup and weakness. A complete metabolic panel at that time showed no evidence of liver transaminitis.

**Figure 1:**
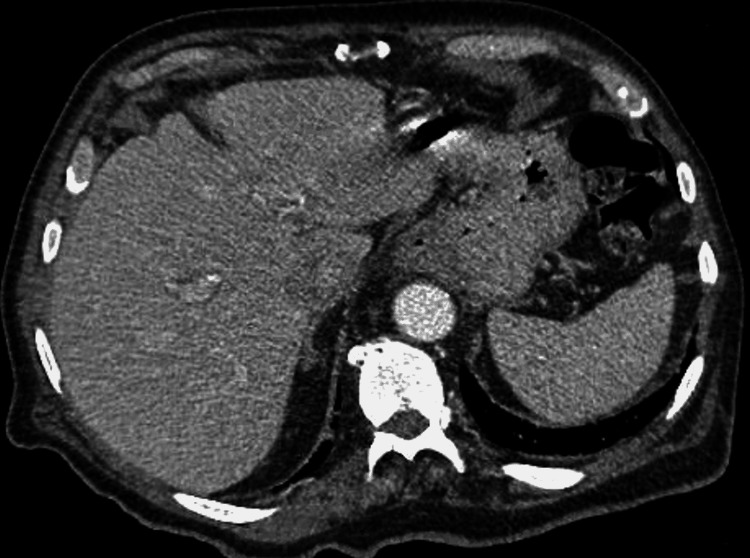
Computed tomography with intravenous contrast of the abdomen showing no acute pathology in the liver.

**Figure 2:**
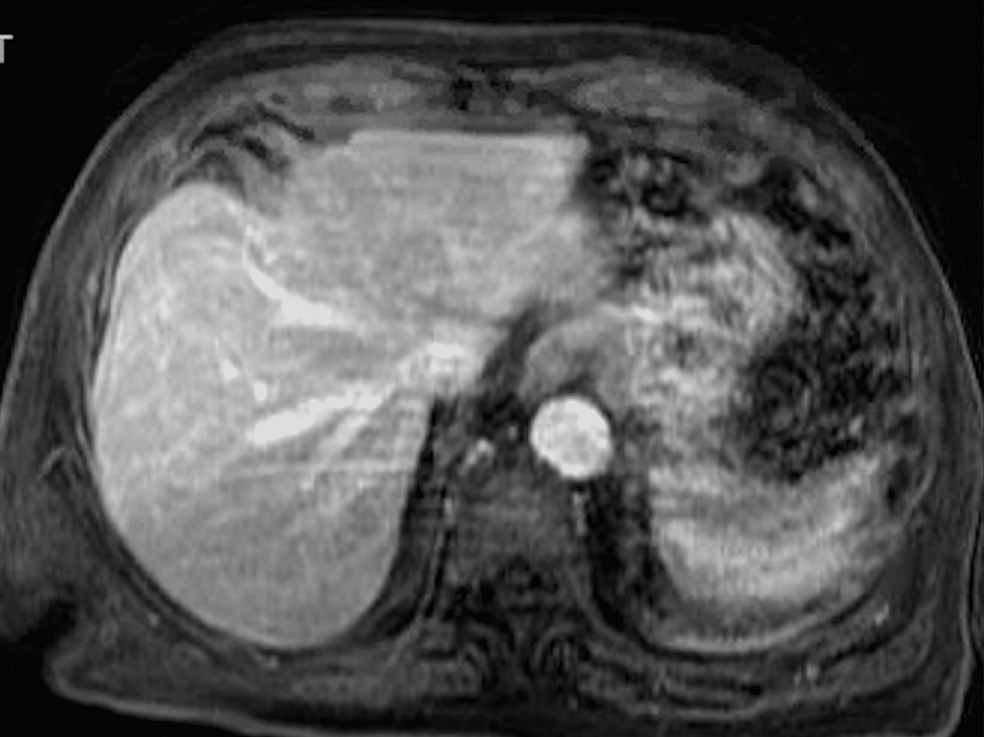
Magnetic resonance cholangiopancreatography (MRCP) of the abdomen showing no acute pathology of the liver.

Soon after admission, acetaminophen 650 mg was given for fever management. The patient was also placed on 40 mg injectable enoxaparin, once daily for deep vein thrombosis prophylaxis. Two days later, lab work started showing evidence of mildly elevated liver enzymes despite the patient not having abdominal complaints. His fever subsided. The aspartate aminotransferase (AST), alanine aminotransferase (ALT), and alkaline phosphatase (ALP) were 99, 96, and 190 µ/L, respectively. On the next day, the patient had an abdominal ultrasound of the right upper quadrant that was negative for any acute abnormalities and showed normal hepatobiliary structures. On the fifth night, the patient's fever was elevated back up to 100.7˚F. Labs showed worsening liver function tests (LFT): AST, ALT, and ALP were 129, 138, and 316 µ/L, respectively. His serum albumin was downtrending and measured at 3.4 g/dL. Acute hepatitis panel was sent that included Hepatitis A/B/C, CMV/EBV, HSV 1/2, *bartonella*, e*hrlichia*, *brucella*, *borrelia burgdorferi*, *coxiella burnetii*, and *rickettsia rickettsia -- *all of which were negative. Computed tomography (CT) of the abdomen with intravenous contrast was negative for acute pathology with regard to the liver and surrounding biliary structures (Figure 1). Magnetic resonance cholangiopancreatography (MRCP) was also done, which showed no acute pathology in the liver or biliary system (Figure 2).

On the sixth day, the patient complained of having chills overnight and some right upper quadrant abdominal tenderness, described as 4/10 at its worst. LFTs continued trending up: AST, ALT, and ALP were 171, 155, and 399 µ/L, respectively. Serum albumin was now 3.1 g/dL. His fever remained high at 100.6˚F in the afternoon. Upon discontinuing enoxaparin, heparin was started at 5,000 units every 12 hours. The seventh day, the patient reported that his abdominal pain and fever had resolved. His LFTs overall peaked that day: AST, ALT, and ALP were 146, 196, and 481 µ/L, respectively. The eighth day, the patient's LFTs started trending down to less than 100s µ/L. The serum albumin returned to normal within three days. The patient had no subjective complaints and his vitals were stable for the next 6 days when he was discharged. A summary of the laboratory values can be found in Table 1.

**Table 1:** A tabulated view of pertinent values over the first eight days of admission.

	Day 1	3	5	6	7	8
AST (reference: 8-48 µ/L	29	99	129	171	146	90
ALT (reference: 7-56 µ/L)	24	96	138	155	196	99
ALP (reference: 44-147 µ/L)	78	190	316	399	481	370
Albumin (reference: 3.4-5.4 g/dL)	3.9	3.6	3.4	3.1	3.3	3.5
Temperature (reference: 97 - 99 ˚F)	100.7	99.2	100.7	100.6	98.9	98.7

## Discussion

Understanding how enoxaparin could potentially have a toxic effect on the liver is vital before considering the COVID-19 vaccine’s role. According to the American Heart Association, proposed mechanisms include direct toxicity, hepatocyte membrane modification, and immune-mediated hypersensitivity reaction [7]. The central theme of all these mechanisms involves inflammation. In terms of the first and second mechanisms, when a cell loses its contents due to its membrane being compromised, apoptosis soon ensues [8]. The first two mechanisms are likely explained by the direct action of enoxaparin on hepatic cells. The third mechanism is more likely to explain how COVID-19 vaccines are likely a culprit in predisposing patients to enoxaparin-mediated liver damage. 

Henceforth, hepatic inflammation secondary to a self-inflicted immune response is where the COVID-19 vaccine comes into play. Interestingly, in the absence of an antigen in the liver, the immune system can still act to harm the liver. One study, explaining the role of systemic viral infections in causing hepatic injury, actually supports this concept [9]. Specifically, it suggested the mechanism occurs due to the T-lymphocytes getting trapped in certain sinusoids and causing regional ischemic damage to the hepatocytes [9]. This study basically paves the way for the possibility of systemic inflammatory states causing direct damage to the liver via T-cells. In fact, COVID-19 vaccines primarily trigger T-cell-mediated immune reactions which can trigger such systemic inflammatory states [10]. Now, this might lead one to connect COVID-19 vaccines primarily to the cause of hepatic injury. However, increased liver enzymes have not been observed in any Moderna COVID-19 vaccine clinical trials to this date; also, no case reports of this event have been identified, in the face of 600 million vaccines being administered [11,12]. This leads us to two plausible conclusions from this case. First, the vaccine potentially could have elicited an inflammatory response that, although not strong enough to cause hepatic necrosis, amplified or catalyzed enoxaparin's effects on the liver. Second, the vaccine potentially had no role in the event of enoxaparin-inducing liver damage. However, one interesting fact that gives more credit to the former possibility is that this patient had received enoxaparin three years ago without any documented adverse reactions. The only difference now is he had received the COVID-19 vaccine shortly prior to this enoxaparin administration.

Additionally, there are certainly odd characteristics in this case that sets our patient apart from the many enoxaparin-related liver injury cases reported. First, our case had a marked fever that spiked after enoxaparin was administered. This is odd and not a normal documented symptom in most cases reported [3]. What makes this more interesting was the fact that this patient already came in with a fever and it had been controlled with the use of acetaminophen. The initial fever most likely adds credibility to the fact that his COVID-19 vaccine played a role in his reaction to the enoxaparin because it is one of the markers of a pre-existing systemic inflammatory state secondary to the vaccine. As discussed before, the vaccine with the exact same variant he had received has been linked to many cases of myocarditis, an inflammation process involving the cardiac muscle tissues [6]. Hence, the vaccine’s ability to stimulate such inflammatory states can potentially act as a mediator for enoxaparin’s effects on the liver. Second, this patient had no acute findings on his CT and MRCP of the abdomen. This could potentially support our former theory delineating how the vaccine itself does not cause the hepatic injury, but rather renders the liver vulnerable due to the body’s inflammatory response to it, as evidenced by the patient’s fever.

Most cases of enoxaparin-induced hepatic injury present asymptomatically which most likely lead to prolonged therapy (given the lack of visual signals indicating sense of urgency); this certainly has the potential to result in necrosis visualized on imaging [3]. However, for our case, the patient presented with symptoms early on, possibly owing to the COVID-19 vaccine’s compounded inflammatory effects, which lead to prompt discontinuation of enoxaparin before necrosis could have resulted.

## Conclusions

Enoxaparin is a commonly used anticoagulant often used to prevent hypercoagulable states. No doubt, its use in the clinical realm has a great amount of success at producing favorable outcomes in hospitalized patients with minimal downside. There is, however, a fair deal of documented hepatocellular damage secondary to its use. This paper serves to establish the Moderna COVID-19 vaccine as a potential mediator in enoxaparin-induced liver injuries. A better understanding of this relationship may prove to be valuable in preventing similar cases and potentially fatal outcomes. Additionally, much remains yet to be elucidated when it comes to sub-acute and long-term complications of drug-vaccine interactions.
